# Hybrid Molecular and Spin Dynamics Simulations for Ensembles of Magnetic Nanoparticles for Magnetoresistive Systems

**DOI:** 10.3390/s151128826

**Published:** 2015-11-13

**Authors:** Lisa Teich, Christian Schröder

**Affiliations:** 1 Bielefeld Institute for Applied Materials Research, Computational Materials Science and Engineering, Bielefeld University of Applied Sciences, P.O. 101113, Bielefeld 33511, Germany; E-Mail: lisa.teich@fh-bielefeld.de; 2 Center for Spinelectronic Materials and Devices, Department of Physics, Bielefeld University, P.O. 100131, Bielefeld 33501, Germany

**Keywords:** hybrid classical spin dynamics and molecular dynamics simulations, nanoparticular GMR effect

## Abstract

The development of magnetoresistive sensors based on magnetic nanoparticles which are immersed in conductive gel matrices requires detailed information about the corresponding magnetoresistive properties in order to obtain optimal sensor sensitivities. Here, crucial parameters are the particle concentration, the viscosity of the gel matrix and the particle structure. Experimentally, it is not possible to obtain detailed information about the magnetic microstructure, *i.e.*, orientations of the magnetic moments of the particles that define the magnetoresistive properties, however, by using numerical simulations one can study the magnetic microstructure theoretically, although this requires performing classical spin dynamics and molecular dynamics simulations simultaneously. Here, we present such an approach which allows us to calculate the orientation and the trajectory of every single magnetic nanoparticle. This enables us to study not only the static magnetic microstructure, but also the dynamics of the structuring process in the gel matrix itself. With our hybrid approach, arbitrary sensor configurations can be investigated and their magnetoresistive properties can be optimized.

## 1. Introduction

The well-known giant magnetoresistance (GMR) effect is commonly used to design extremely sensitive sensors that respond to external magnetic fields with changes in their electrical resistance. The GMR effect was originally discovered in magnetic multilayer systems [1,2]. Later it was also found for systems that contain magnetic grains in metallic matrices [3,4]. Recently, it could be shown that systems made of magnetic particles that are immersed in conductive gel matrices show GMR behavior as well. Besides achieving very high GMR effect amplitudes, the use of gel matrices opens up the perspective of printable, low-cost, magnetoresistive sensor devices such as sensors for the detection of biomolecules [5,6,7]. The sensor characteristics directly depend on the microscopic behavior of the magnetic particles in the gel matrix, *i.e.*, on the dynamics of the magnetic moments of the particles in combination with their local positions and motion in the gel. Since such microscopic information cannot be directly extracted from experiments, numerical simulations have to be taken into account. In order to simulate the magnetodynamics of an ensemble of magnetic nanoparticles that are embedded in a conductive gel matrix, two different simulation methods have to be combined in order to simultaneously consider both the magnetic and translational degrees of freedom of the magnetic particles. In order to do so, one has to consider the time scales of the two classes of degrees of freedom as well as a method to control the temperature. Here, we present a hybrid approach that couples molecular dynamics and classical spin dynamics methods so as to simulate the structuring process of spherical magnetic nanoparticles in a viscous surrounding medium. In contrast to other hybrid molecular and spin dynamics algorithms presented in the literature [8,9,10,11,12,13,14], a purely classical approach is followed here. Moreover, the known methods follow completely different approaches and, therefore, instead of a coupling of the spin and mechanical degrees of freedom, couplings of spin and other degrees of freedom are realized. For example, in [8,9], a hybrid method is presented that covers the spin and electronic degrees of freedom whereas in [13] a coupling between spin and lattice dynamics is introduced. In addition to that, we follow a coarse grain approach, whereas the other methods pursue an atomistic model. Our hybrid molecular and spin dynamics approach has been introduced in [6] as one part of a simulation tool chain. Therein, a basic description is given without computational details. Here, we provide a complete and comprehensive derivation of our approach. Starting from the general approach, we briefly describe both numerical methods separately. Subsequently, we present the theoretical foundations and introduce our hybrid algorithm. Finally, we show one exemplary simulation run for didactic purposes.

## 2. General Approach

In this paper we assume magnetically interacting nanoparticles that are free to move in a viscous medium. In order to simulate their behavior one has to solve the following sets of coupled equations of motion:

(1) $$m_{i}\frac{dv_{i}\left(t\right)}{dt}=-\nabla_{r_{i},S_{i}}{ℋ}_{DD}\left(r_{1},\ldots ,r_{N},\;S_{1},\;\ldots ,\;S_{N}\right)-F_{visc}\left(\;v_{1},\;\ldots ,v_{N}\right)-\nabla_{r_{i}}{ℋ}_{WCA}$$

(2) $$\frac{dr_{i}\left(t\right)}{dt}=v_{i}\left(t\right)$$

(3) $$\hbar \frac{\partial S_{i}}{\partial t}=\;H_{eff}\left(r_{1},\ldots ,r_{N},\;S_{1},\;\ldots ,\;S_{N}\right)\times S_{i}-\lambda \;\left(H_{eff}\left(r_{1},\ldots ,r_{N},\;S_{1},\;\ldots ,\;S_{N}\right)\times S_{i}\right)\times S_{i}$$

Here, the first and second set of equations is used to compute the velocity $v_{i}$ and position $r_{i}$ of each particle in the ensemble. The force $F_{visc}$ describes the viscous drag of the surrounding fluid. The term ${ℋ}_{DD}$ is the magnetic potential due to the dipole-dipole interaction among all particles which depends on the positions of the particles as well as on the orientations of their magnetic moments $S_{i}$. The last term of the first equation describes the repulsive force due to the Weeks-Chandler-Andersen potential which is necessary in order to simulate hard sphere dynamics. The third set of equations calculates the orientations of the magnetic moments $S_{i}$. Here, $H_{eff}$ denotes an effective magnetic field which is generated by the magnetic interactions between the particles. The last term is the so-called Landau-Lifshitz damping which is non-conservative and used to find the appropriate magnetic low energy configurations for a given particle arrangement (see below). Here, λ describes the friction and can be adjusted in order to reach a sufficient numerical stability. In summary, there are three important potential or force contributions. These contributions are described in detail in Section 2.1.2.

Although the equations of motion (1–3) are fully coupled by the dipole-dipole potential $\;{ℋ}_{DD}$ one can show that under certain conditions (see below) it is not necessary to solve these equations simultaneously but to separate their calculation leading to a unidirectional consecutive approach. In this approach, the magnetic configuration, *i.e.*, the orientation of the magnetic moments, for a given initial nanoparticle arrangement is calculated first. This magnetic configuration creates magnetic forces that act on the particles inducing their subsequent motion which in turn changes the orientation of the magnetic moments and so on, resulting in a hybrid approach. The advantage of our hybrid approach is that the calculation of the magnetic low energy configuration and the calculation of the resulting translational motion can be executed separately by two different simulation methods which are explained in the following.

### 2.1. Molecular Dynamics Equations of Motion

Classical molecular dynamics (MD) provides a method to simulate the motion of atoms, molecules or particles due to interactions between the particles or interactions with external fields by solving the following set of equations of motion:

(4) $${\text{m}}_{\text{i}}\frac{\partial v_{\text{i}}}{\partial \text{t}}=\nabla_{r_{\text{i}}}\text{Φ}$$

(5) $$\frac{\partial r_{\text{i}}}{\partial \text{t}}=v_{\text{i}}\left(\text{t}\right)$$

In contrast to *ab initio* methods which are based on quantum mechanical principles, classical MD is based on classical potential formulations Φ from which the according forces on the particles are derived. MD can be applied to a broad range of fields, e.g., theoretical physics, physical chemistry, biophysics and materials science. In this work, we have extended the open-source code HOOMD-blue [15,16,17] with the necessary customizations as given below.

#### 2.1.1. Integration of the Equations of Motion

Particles in classical MD systems obey the laws of Newtonian mechanics. There exists a multitude of standard algorithms to integrate the Newtonian equations of motion (4,5), *i.e.*, the Euler algorithm, different types of Runge-Kutta schemes and Verlet-type methods [18]. Here, we use one of the most important algorithms for the integration of the equations of motion, namely the Velocity Verlet algorithm which is a variant of the well-known Leapfrog algorithm. In contrast to other integration algorithms, positions and velocities are calculated for the same steps in time which is a great advantage of the Velocity Verlet algorithm. Moreover, this algorithm exhibits good energy conservation and is time-reversible. Equations (6) and (7) show how the positions $r_{i}$ and velocities $v_{i}$ are calculated for each particle [18,19]:

(6) $$r_{i}\left(t+\Delta t\right)=r_{i}\left(t\right)+v_{i}\left(t\right)\cdot \Delta t+\frac{F\left(t\right)}{2m_{i}}\Delta t^{2}$$

(7) $$v_{i}\left(t+\Delta t\right)=v_{i}\left(t\right)+\frac{F\left(t+\Delta t\right)+F\left(t\right)}{2m_{i}}\Delta t$$

#### 2.1.2. Force Calculation

The forces that act on the particles due to particle-particle or particle-field interactions have to be calculated in every time step of an MD simulation. The calculation of particle-particle interactions is extremely time-consuming because it is of order $N^{2}$. In some cases the computational effort can be reduced by means of neighbor list or cell computation approaches [18]. In our case we assume that the magnetic nanoparticles solely interact by magnetic dipole-dipole interaction which exhibits particular long-ranged characteristics. In order to preserve this long-range behavior, methods to reduce the computational effort cannot be applied because all of these approaches utilize a potential cut-off. Therefore, the full calculation of all pairwise contributions is used here which provides correct forces for short and long distances between the particles [18,19]. In order to model magnetic nanoparticles that move due to their magnetic interactions while being immersed in a viscous matrix, three contributions to the net force must be considered:

##### Magnetic Dipole-Dipole Interaction

Every magnetic particle in the system under consideration creates a magnetic dipole field which exerts a torque on the dipole field of every other particle. The pairwise potential contributions can be calculated according to the following Equation (8):

(8) $${ℋ}_{DD,i,j}=-\frac{{µ}_{0}}{4\pi r_{ij}^{3}}\left[3\left(S_{i}\cdot {\hat{r}}_{ij}\right)\left(S_{j}\cdot {\hat{r}}_{ij}\right)-S_{i}\cdot S_{j}\right]$$

**Figure 1:**
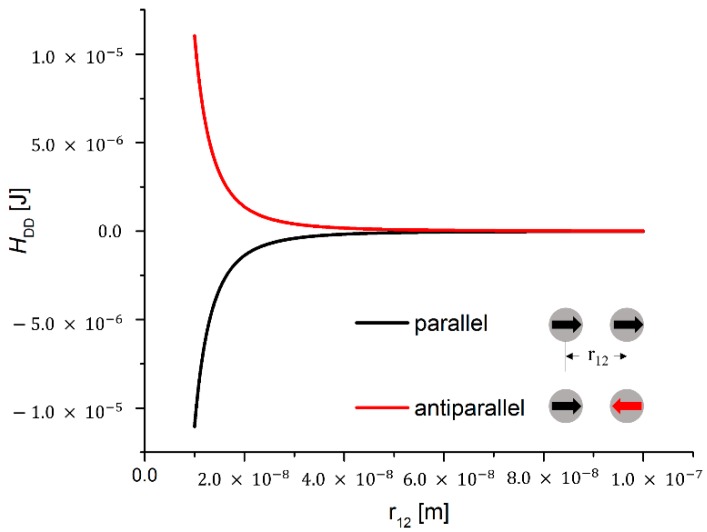
Magnetic dipole-dipole energy curves for the case of two interacting cobalt particles with diameters of 10 nm. The magnetic dipole-dipole energy is plotted *vs.* the center-to-center distance of the particles. For the case of particles with magnetic moments aligned in parallel, negative energy values are obtained (black curve), resulting in particle attraction. In contrast to this, for an antiparallel alignment of the magnetic moments, positive energy values are obtained (red curve), resulting in particle repulsion. It becomes apparent that the magnetic dipole-dipole potential contribution cannot be cut off due to its long-range character over a distance of several particle diameters even for small particles.

Therein, $S_{i}$ and $S_{j}$ represent the magnetic moments of two particles *i* and *j* while ${\hat{r}}_{ij}$ describes the unit vector that connects the centers of the particles. The distance between the centers of the particles is given by $r_{ij}$. The magnetic dipole-dipole interaction causes a force that acts along ${\hat{r}}_{ij}$. Depending on the relative orientations of the magnetic moments of the particles, it can be attractive or repulsive. An example of the distance dependence of the magnetic dipole-dipole interaction energy of two interacting cobalt particles with diameters of 10 nm is depicted in Figure 1. It is evident that a well-defined cut-off radius does not exist due to the long-range character of the interaction. 

##### Hard Particle Approach

In general, point masses are used in MD simulations. However, this means for the particle positions that they can become identical or that distances $r_{ij}$ can become infinitesimally small, *i.e.*, $r_{ij}\to 0$. In such cases the dipole-dipole potential Equation (8) diverges which would lead to numerical instabilities and unphysical behavior. In order to avoid this, potential functions for molecular dynamics simulations of hard particles have to be used which are based on a combination of attractive and repulsive contributions. The so-called Weeks-Chandler-Andersen (WCA) potential provides a force-shifted formulation of the well-known Lennard-Jones potential which provides the required short-range repulsion [20]. Above a distance of two particle diameters $r_{ij}\;\geq \;2^{\frac{1}{6}}\sigma$, the WCA potential is cut off. Below this distance, a Lennard-Jones type repulsion is modeled:

(9) $${ℋ}_{WCA}=\{\begin{array}{ll} 4\varepsilon \left[{\left(\frac{\sigma }{r_{ij}}\right)}^{12}-{\left(\frac{\sigma }{r_{ij}}\right)}^{6}\right]+\varepsilon , & ifr_{ij}<2^{\frac{1}{6}}\sigma \\ 0, & ifr_{ij}\geq 2^{\frac{1}{6}}\sigma \end{array}$$

**Figure 2:**
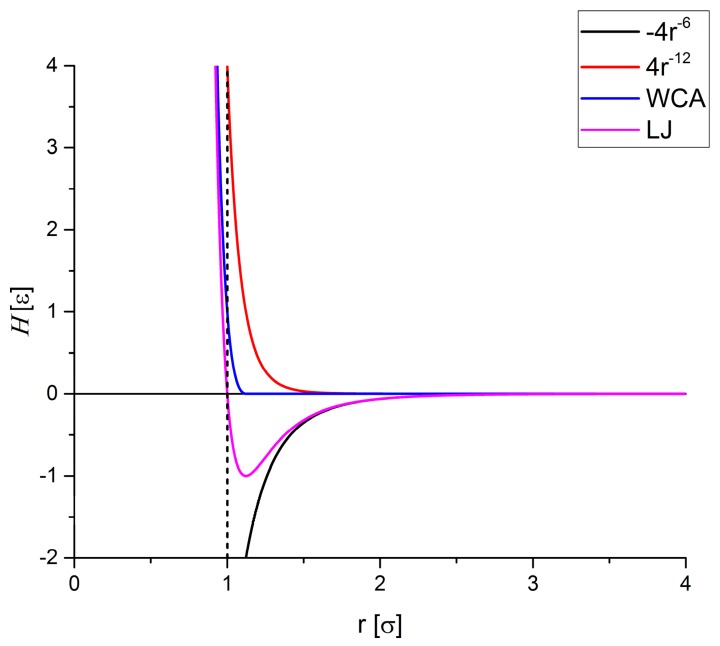
Comparison of attractive and repulsive Lennard-Jones (LJ) potential (magenta) and short-range repulsive Weeks-Chandler-Andersen (WCA) potential (blue) together with the dominating contributions $4\times r^{-12}$ (red) and $-4\times r^{-6}$ (black). Here, the respective potential ℋ which is given in units of energy is plotted as a function of the distance between two fictitious particles r given in units of distance.

Here, the parameter σ is given in units of distance and ε is given in units of energy. Figure 2 illustrates the WCA potential for the case σ = ε = 1.

##### Stokes Drag

To take into account the surrounding medium that affects the motion of the particles due to its viscosity, a third force contribution must be included. Stokes’ law describes the force that acts on a small spherical particle with a radius $r_{p}$ and with a given velocity $v_{p}$ that is embedded in a viscous medium with kinematic viscosit $\;\eta_{m}$. The resulting drag force acts in the direction opposite to the particle’s motion and it is proportional to its velocity and the viscosity of the medium.

(10) $$F_{visc}=6\pi r_{p}\eta_{m}v_{p}$$

### 2.2. Classical Spin Dynamics Simulations

Classical spin dynamics is a proven method for the numerical analysis of the static and dynamic thermodynamic properties of microscopic and mesoscopic ensembles of classical magnetic moments. Here, we use a spin dynamics algorithm that involves solving the Landau-Lifshitz equation. A Langevin approach is utilized to model the contact to an external heat bath. Moreover, the particles are assumed to be homogeneously magnetized and therefore modeled by a single magnetic moment. A detailed description of the algorithm is presented in [21].

Spin Equations of Motion: A magnetic moment $S_{i}$ that is subject to an external magnetic field precesses around the external field direction. In a classical picture this precession is given by:

(11) $$\hbar \frac{\partial S_{i}}{\partial t}=\frac{\partial ℋ}{\partial S_{i}}\times S_{i}$$

where ℋ is the Hamiltonian that contains the magnetic interactions, *i.e.*, exchange, Zeeman, anisotropy and dipole-dipole interactions. In this paper we only consider the dipole-dipole interaction. For systems of magnetic nanoparticles that are exposed to a homogeneous external magnetic field, the Zeeman contribution has to be included as well [21]. It is convenient to use the partial derivative of the Hamiltonian *H*, *i.e.*, $\frac{\partial ℋ}{\partial S_{i}}=H_{eff}$ which is the effective magnetic field $H_{eff}$ caused by all interactions that act on the spin under consideration. Equation (11) gives rise to a conservative dynamics. In order to be able to calculate low energy configurations, Equation (11) needs to be extended by a damping term which allows the system to relax towards its ground state. This is commonly done by adding a damping term as proposed by Landau and Lifshitz:

(12) $$\frac{\partial S_{i}}{\partial t}=\;\frac{1}{\hbar }H_{eff}\times S_{i}-\lambda \;\frac{1}{\hbar }\left(H_{eff}\times S_{i}\right)\times S_{i}$$

In doing so, the spins relax until they are oriented parallel to the effective field direction. The damping force which is proportional to the positive damping constant λ, acts in the direction of the effective field [21]. By solving Equation (12) it is not guaranteed that the system’s ground state is found. In fact, for more complex systems the algorithm “gets stuck” in the local energy minimum that is first reached in phase space and there is no way to get out of this. In order to overcome this problem one can extend Equation (12) by a heat bath coupling. Here, we use a Langevin approach in which the heat bath acts on the system by means of stochastic forces. Hence, the classical spins fluctuate due to forces that are driven by the temperature of the heat bath and “kick” the system out of local minima. The fluctuations are introduced into the equations of motion by the additional term:

(13) $$f_{i}\left(t\right)\times S_{i}$$

with $f_{i}\left(t\right)$ representing the undirected fluctuations that have a white noise characteristic. As a result one obtains the following stochastic differential equation of motion for the spin degrees of freedom:

(14) $$\frac{\partial S_{i}}{\partial t}=\;\frac{1}{\hbar }H_{eff}\times S_{i}-\lambda \;\frac{1}{\hbar }\left(H_{eff}\times S_{i}\right)\times S_{i}+f_{i}\left(t\right)\times S_{i}$$

In order to integrate Equation (14) we have used a fourth-order Runge-Kutta scheme as proposed by Milstein and Tretyakov [21,22].

## 3. Hybrid Molecular and Spin Dynamics Simulations

As pointed out in Section 2, the simulation of interacting magnetic nanoparticles in a viscous medium generally requires solving the system of coupled differential Equations (1)–(3) for the translational and magnetic degrees of freedom. However, this is only necessary if the time scales of the dynamics for the translational degrees of freedom and magnetic degrees of freedom are of the same order. If this is not the case the magnetic and translational degrees of freedom can be treated separately, leading to a simple coupling scheme reminiscent of the method proposed by Dünweg and Ladd for the numerical simulation of colloidal dispersions [23]. In order to validate this assumption for our problem, the relaxation times of the two types of degrees of freedom are determined in the following.

### 3.1. Translational Relaxation Time 

In order to determine the time scale of the translational relaxation of a particle of spherical shape, the configurational relaxation time [23] must be evaluated. The configurational relaxation time, or Brownian relaxation time, is the time that the particle needs to diffuse across its own radius and can be evaluated according to:

(15) $$\tau_{cr}=\frac{\eta R^{3}}{k_{B}T}$$

Here, η represents the kinematic viscosity of the matrix material, R is the particle diameter, $k_{B}$ is the Boltzmann constant and T represents the temperature [24].

### 3.2. Magnetic Relaxation Time 

The magnetization of a colloidal magnetic particle relaxes due to two different mechanisms. First, the relaxation can occur via rotation of the particle in the liquid. The characteristic time associated with this mechanism is called Brownian rotational diffusion time and can be calculated according to Equation (16) with V representing the particle volume:

(16) $$\tau_{B}=\frac{3V\eta }{k_{B}T}$$

The second mechanism is the so-called Néel rotation where the magnetization of the particle relaxes due to the coherent rotation of atomic magnetic moments within the particle. For a uniaxial, single-domain, ferromagnetic particle, the associated time can be calculated by:

(17) $$\tau_{N}=\frac{1}{f_{0}}\text{exp}\left(\frac{KV}{k_{B}T}\right)$$

where, K represents the magnetic anisotropy constant of the particle material and $f_{0}$ is a characteristic frequency [25].

**Figure 3:**
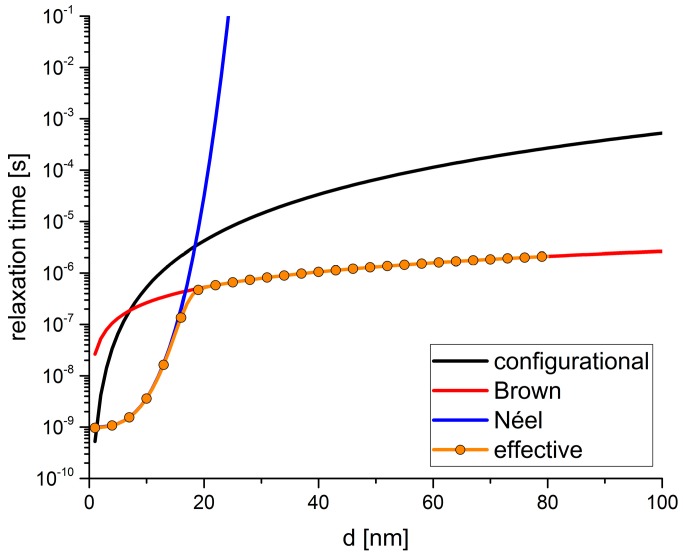
Relaxation times of the translational and the magnetic degrees of freedom of ensembles of magnetic cobalt particles that are immersed in an agarose matrix. The translational relaxation is dominated by the configurational relaxation time (black) whereas the magnetic relaxation can be dominated by Brownian rotation (red) or Néel rotation (blue). Within our algorithm, a difference between Brown and Néel relaxation cannot be addressed and hence, both mechanisms result in a change of the magnetic moment orientation without changing the orientation of the particle itself. Hence, the configurational relaxation time must simultaneously be compared to the magnetic relaxation times that are combined to the effective magnetic relaxation time (orange) according to Equation (18). There is a significant difference between the magnetic and mechanical relaxation over all particle diameters except for very small particle and one small window around the particle diameter of 20 nm.

As shown in Figure 3, for small particles the Néel relaxation dominates whereas for larger particles, the Brown relaxation dominates. In addition to that, a transition regime can be identified around particle diameters of about 20 nm. Because both mechanisms contribute to the relaxation behavior of ensembles of magnetic particles, an effective relaxation time can be defined according to the following Equation [26]:

(18) $$\tau_{eff}=\frac{\tau_{B}\tau_{N}}{\tau_{B}+\tau_{N}}$$

### 3.3. Comparison of Translational and Magnetic Relaxation Times 

In order to check whether the translational and the magnetic degrees of freedom can be separated, the time scales have been evaluated for cobalt particles with diameters up to 100 nm that are immersed in agarose gel matrices with a common concentration. In our current algorithm we do not consider a difference between Brownian and Néel rotation but will address this in further studies. Thus, the relevant time scales are set by the configurational relaxation time $\tau_{cr}$ and both the Néel rotation diffusion time $\tau_{N}$ and the Brownian relaxation time $\tau_{B}$ that are combined to the effective relaxation time $\tau_{eff}$. For $\tau_{N}$ we have chosen a characteristic frequency of $f_{0}=\;10^{9}\;\text{Hz}$ and an anisotropy constant of $K=10^{5}\;\text{J}\cdot {\text{m}}^{-3}$ [6,27]. For $\tau_{cr}$we have used η=0.017  Pa·s for the kinematic viscosity of the gel matrix which corresponds to a 2% agarose gel matrix as it is used in [5,7]. The results are shown in Figure 3. Here, it becomes apparent that the Néel relaxation time is very small for small particle diameters and rapidly increases with increasing particle diameter. In contrast to that, the Brown relaxation time slowly increases with increasing particle diameter. By combining both relaxation times according to Equation (18), the relevant effective magnetic relaxation time is obtained. A comparison of this effective relaxation time and the mechanical, configurational relaxation time shows that a separation of the time scales exists for all particle diameters except for very small diameters up to around 3 nm. The smallest difference between the time scales for larger particles can be identified around the transition regime for particle diameters of about 20 nm. Nevertheless, even at this point, the time scales are clearly separated by at least one order of magnitude.

### 3.4. Hybrid Molecular and Spin Dynamics Coupling Scheme

**Figure 4:**
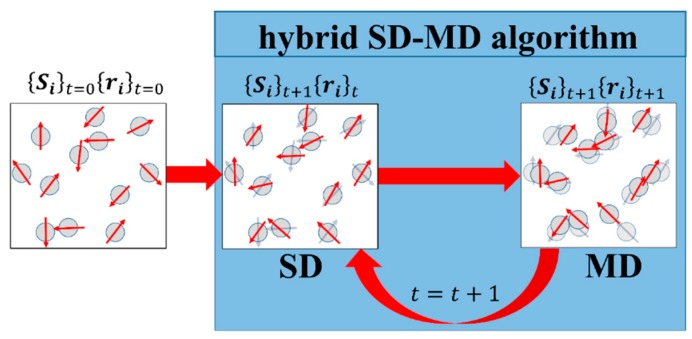
Schematic representation of the hybrid algorithm that couples spin dynamics (SD) and molecular dynamics (MD) simulations in order to calculate trajectories of magnetic nanoparticles that interact via magnetic dipole-dipole interaction. Starting from a random initial configuration ${\left\{S_{i}\right\}}_{\text{t}=0}{\left\{r_{i}\right\}}_{\text{t}=0}$, the first magnetic low energy state is calculated by means of SD. Therein, the particle positions are retained whereas the orientations of the magnetic moments are changed to ${\left\{S_{i}\right\}}_{\text{t}+1}$. The new orientations together with the fixed particle positions are then passed over to the MD algorithm. Within MD, the magnetic moment orientations are maintained while the particles are moved forward for a small step in time due to the forces that act on the particles. The resulting particle positions ${\left\{r_{i}\right\}}_{\text{t}+1}$are then passed over to SD again in order to calculate the magnetic low energy state of the new configuration. This procedure is repeated until a predefined number of time steps is reached.

In our hybrid coupling scheme which is depicted in Figure 4, we start from an initial random magnetic moment configuration ${\left\{S_{i}\right\}}_{0}$. In principle, the magnetic moments are three-dimensional vectors. However, due to their initial coplanar arrangement the dipole-dipole interaction leads to a coplanar orientation of the magnetic moments as well. The initial particle positions ${\left\{r_{i}\right\}}_{0\;}$ are taken from experimental data, e.g., microscopic images. Using ${\left\{S_{i}\right\}}_{0}$ and ${\left\{r_{i}\right\}}_{0}$ the magnetic moment orientations are relaxed towards a low energy state by means of spin dynamics (SD) simulations. Hence, the magnetic moment orientations are updated while the particle positions are fixed. This procedure leads to a new set of data, *i.e.*, ${\left\{S_{i}\right\}}_{1}$ and ${\left\{r_{i}\right\}}_{0}$ which is used as input data for the subsequent MD simulation. As presented in Section 2.1, the MD algorithm first calculates the net force that acts on every particle according to Equation (1). In a second step, the equations of motion are integrated over a time step Δt according to Equations (6) and (7) leading to the new positions ${\left\{r_{i}\right\}}_{1}$ while the magnetic moment orientations are kept unchanged. The new set of data ${\left\{S_{i}\right\}}_{1}$ and ${\left\{r_{i}\right\}}_{1}$ is used as input data for the subsequent SD simulation. As shown in Figure 4, this process is repeated until a predefined number of total time steps is reached. 

### 3.5. The Role of Temperature

In general, both methods MD and SD work in the canonical ensemble, *i.e.*, the temperature is kept constant during the simulation. In order to avoid conflicts between the two heat baths, the temperature of the SD part of the simulation is set to 0 K whereas the MD simulations are performed at room temperature. As explained in [28], it is common practice for the simulation of macromolecules in solution to couple separate thermostats to the subsystems. This practice is based on ab initio methods such as the Car-Parrinello approach [29] where the nuclear and electronic subsystems are separated dynamically. Whereas the slow nuclei are connected to a “physical” temperature, the fast electronic degrees of freedom are assumed to be linked to a “fictitious” temperature. According to [30], the “cold” electronic subsystem is assumed to be close to its instantaneous minimum energy. In analogy to that, here, we assume that the “cold” magnetic degrees of freedom are close to their minimum energy configuration. Hence, it is justified to set the temperature of the SD simulation to 0 K. However, as pointed out in Section 2.2 we use a finite spin bath temperature during the SD simulation step in order to find configurations of lowest energy. 

Here, slowly cooling down from a high initial temperature to 0 K during the simulation leads in most cases to configurations of lowest energy. If this method is not applicable we use more sophisticated strategies that are based on experimental demagnetization protocols which involve rotating and damped external magnetic fields for finding lowest energy configurations in spin ice systems [27]. 

### 3.6. Calculation of Qualitative GMR Curves

We use our hybrid SD-MD algorithm in order to predict the particle arrangements in the liquid state of the gel which corresponds to the experimental preparation stage. After the particle-gel mixture is put on a substrate, the structuring process with or without an external magnetic field takes place. Afterwards, the gel matrix is dried out and the particle structure is preserved. For the application in a sensor device, the actual measurement takes places in the solid state of the gel. Therefore, the magnetoresistive properties in the solid state of the gel are of great importance. In order to determine the magnetoresistive properties in a qualitatively way we use the SD algorithm alone which corresponds to the solid state of the gel with mechanically frozen particles. By means of further spin dynamics simulations, magnetization curves can be calculated. Finally, qualitative GMR curves can be calculated from the magnetization curves by:

(19) $$GMR=A_{GMR}\left[1-{\left(\frac{M}{M_{S}}\right)}^{2}\right]$$

Therein, GMR is the GMR effect given in percent, $A_{GMR}$ is the GMR effect amplitude that has to be extracted from experimental data or further numerical investigation [6,31], M is the magnetization and $M_{S}$ represents the material’s saturation magnetization. The resulting GMR curves lead to a qualitative estimate of the magnetoresistive properties of a particular particle-gel combination which provides useful information for the actual sensor development.

## 4. Application: Magneto-Dynamics of Interacting Magnetic Nanoparticles in Gel Matrices for the Efficient Design of Magnetoresistive Systems

The hybrid SD-MD algorithm can be used for the investigation of the magneto-dynamics of magnetic nanoparticles that are used in combination with conductive gel matrices to build nanoparticular magnetoresistive sensor devices. As presented in [6], this type of sensor can be used for the detection of biomolecules. Therefore, the GMR sensor is covered with antibodies that are chosen for a specific analysis purpose, *i.e.*, the biomolecules under consideration in a sample solution bind to the sensor surface. In order to utilize the GMR effect for the measurement of the biomolecule concentration, magnetic marker particles have to be used. The binding of the marker particles to the biomolecules is realized by using antibodies as well. As a result, the stray field of the magnetic marker particles changes the initial configuration of the magnetic particles and therefore the magnetoresistive properties. Thus, a change in the electrical resistance of the structure can be measured. The sensitivity of such a sensor is related to its magnetoresistive properties which are defined by the microstructure, *i.e.*, the arrangement of the magnetic nanoparticles. Here, we show simulations of the structuring process of an ensemble of magnetic nanoparticles in a viscous matrix. In addition to that, we show how the magnetoresistive properties can be obtained from simulated magnetization curves.

### 4.1. Model System

**Figure 5:**
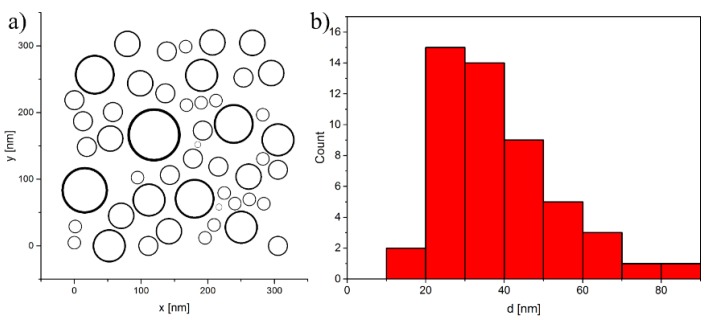
Model structure consisting of 50 cobalt nanoparticles with diameters in the range between 10 nm and 80 nm (**a**). The particles are distributed over an area of 350 nm × 350 nm. The size distribution (**b**) is taken from experimental studies on particle-based GMR sensors [5,7].

In general, the hybrid SD-MD algorithm presented in Section 3 can be applied to arbitrarily large systems and is only limited by computational resources. In order to demonstrate its applicability we have modeled a section of a real system made of cobalt nanoparticles [5,7] with a broad size distribution as shown in Figure 5. It can be considered as a small region of a magnetoresistive sensor structure. Here, 50 cobalt particles with diameters in the range between 10 nm and 80 nm are distributed over an area of 350 nm × 350 nm. The particles are immersed in a 4% agarose gel matrix, *i.e.*, we assume a viscous surrounding medium with a kinematic viscosity of η=0.11 Pa·s [7]. 

### 4.2. Structuring Process

**Figure 6:**
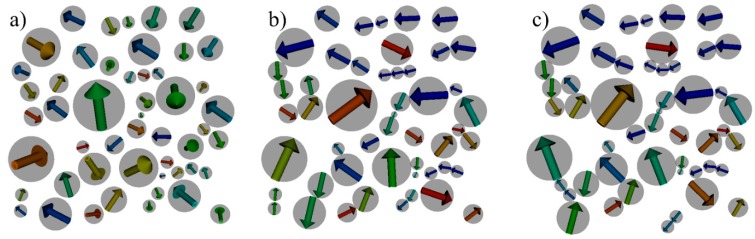
Results of a hybrid SD-MD simulation run of the model structure shown in Figure 5 in zero external magnetic field. The magnetic particles are shown in grey whereas the effective magnetic moments of the particles are represented by colored arrows. The colors of the arrows represent the horizontal, in-plane component of the magnetic moment. Starting from a random initial magnetic configuration with coplanar particle positions and random three-dimensional effective magnetic moment orientations (**a**), a stable final structure is reached after 3 × 10^7^ hybrid SD-MD time steps (**c**). An intermediate configuration after 3 × 10^6^ steps in time is shown in (**b**).

The structuring process of the cobalt nanoparticle ensemble has been simulated by our hybrid SD-MD method. In order to reach a stable end configuration, a MD run with a total number of 3 × 10^7^ time steps and time step length Δt= 1 × 10^−14^ (in reduced Lennard-Jones units) has been carried out with one SD run every 10^4^ time steps. Every SD run took 1 × 10^4^ time steps with a step length of Δt= 1 × 10^−15^ s. Due to significant changes of the magnetic moment orientations in the beginning of the simulation, the structural changes are much larger in the beginning and become slower in the end of the simulation. The simulation results are shown in Figure 6. In agreement with previous theoretical and experimental studies [5,6,7], the self-assembly process of the cobalt particles leads to a configuration that consists of parallel aligned areas, antiparallel chains and particle islands that contain magnetic moment vortices.

### 4.3. Estimation of GMR Properties

**Figure 7:**
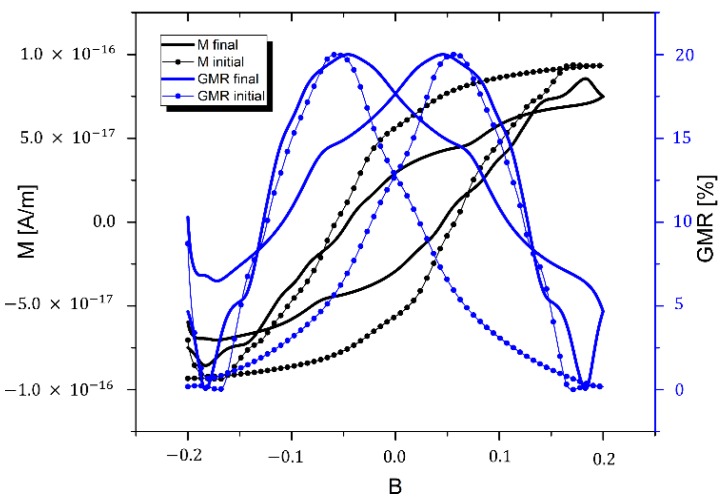
Simulated magnetization curves of the final configuration of the hybrid SD-MD simulation presented in Figure 6c (black line) and, for comparison, the magnetization curve of the initial configuration shown in Figure 6a (black dots). A rough estimate of the GMR properties of the system has been calculated by means of equation 12 (initial configuration with blue dots and final configuration with blue line) for an effect amplitude $A_{GMR}$ of 20% according to previous experimental investigations [5,6,7].

As presented above, the hybrid SD-MD algorithm considers the particle-gel systems in the liquid states of the gel. By switching off the MD algorithm, the magnetization curve of the system in the solid state of the gel can be calculated by means of spin dynamics simulations alone. The resulting magnetization curves and the GMR calculated using Equation (19) are shown in Figure 7. Furthermore, we show in Figure 7, the corresponding magnetization and GMR curve of the initial particle configuration in order to emphasize the difference of the magnetic properties of the initial configuration and the final configuration that results from the hybrid molecular and spin dynamics trajectory calculation. The GMR effect amplitude is assumed to be 20% in agreement with previous experimental investigations on similar structures [5,6,7].

The GMR curves can only be considered a first rough estimate for the magnetoresistive properties of the complete structure since we have only calculated a small section of it. This section contains a few rather large cobalt particles with large magnetic moments that dominate the total magnetization calculation. As a result of this the curves are rather bumpy which would disappear if one simulates larger sections. Nevertheless, even by simulating small sections of a sensor structure different particle materials, size distributions and gel matrices can be investigated in order to optimize parameters for future GMR sensor systems.

## 5. Conclusions/Outlook

GMR sensor devices based on magnetic nanoparticles immersed in conductive gel matrices show promising features for the development of disposable low-cost devices for the detection of biomolecules. In order to optimize the magnetoresistive properties of such sensors it is necessary to know detailed information about the magnetic microstructure. Since this information is not accessible by experimental techniques we provide a novel simulational approach based on hybrid spin dynamics and molecular dynamics methods that allows one to simulate the structuring process of the particles in the liquid state of the gel. Moreover, we have shown that our approach can be used to characterize the GMR properties of a sensor structure. Future enhancements of the hybrid algorithm will include inhomogeneous magnetic fields for the structuring process as well as non-spherical particles such as nanorods and nanocubes. 

## References

[1] Binasch G., Grünberg P., Saurenbach F., Zinn W. (1989). Enhanced magnetoresistance in layered magnetic structures with antiferromagnetic interlayer exchange. Phys. Rev. B.

[2] Baibich M.N., Broto J.M., Fert A., van Dau F.N., Petroff F., Etienne P., Crouzet G., Friederich A., Chazelas J. (1988). Giant Magnetoresistance of (001)Fe/(001)Cr Magnetic Superlattices. Phys. Rev. Lett..

[3] Xiao J.Q., Jiang J.S., Chien C.L. (1992). Giant magnetoresistance in nonmultilayer systems. Phys. Rev. Lett..

[4] Berkowitz A.E., Mitchell J.R., Carey M.J., Young A.P., Zhang S., Spada F.E., Parker F.T., Hutten A., Thomas G. (1992). Giant magnetoresistance in heterogeneous Cu-Co alloys. Phys. Rev. Lett..

[5] Meyer J., Rempel T., Schäfers M., Wittbracht F., Müller C., Patel A.V., Hütten A. (2013). Giant magnetoresistance effects in gel-like matrices. Smart Mater. Struct..

[6] Teich L., Kappe D., Rempel T., Meyer J., Schröder C., Hütten A. (2015). Modeling of Nanoparticular Magnetoresistive Systems and the Impact on Molecular Recognition. Sensors.

[7] Rempel T., Meyer J., Teich L., Gottschalk M., Rott K., Kappe D., Schröder C., Hütten A. Giant magnetoresistance effects in gel-like matrices: Comparing experimental and theoretical data.

[8] Antropov V.P., Katsnelson M.I., van Schilfgaarde M., Harmon B.N. (1995). *Ab Initio* spin dynamics in magnets. Phys. Rev. Lett..

[9] Antropov V.P., Katsnelson M.I., Harmon B.N., van Schilfgaarde M., Kusnezov D. (1996). Spin dynamics in magnets: Equation of motion and finite temperature effects. Phys. Rev. B.

[10] Omelyan I.P., Mryglod I.M., Folk R. (2001). Algorithm of molecular dynamics simulations of spin liquids. Phys. Rev. Lett..

[11] Omelyan I.P., Mryglod I.M., Folk R. (2001). Molecular dynamics simulations of spin and pure liquids with preservation of all the conservation laws. Phys Rev. E.

[12] Omelyan I.P., Mryglod I.M., Folk R. (2002). Construction of high-order force-gradient algorithms for integration of motion in classical and quantum systems. Phys. Rev. E.

[13] Ma P.-W., Woo C.H. (2008). Large-scale simulation of the spin lattice dynamics in ferromagnetic iron. Phys. Rev. B.

[14] Thibaudeau P., Beaujouan D. (2012). Thermostatting the atomic spin dynamics from controlled demons. Phys. A.

[15] Anderson J.A., Lorenz C.D., Travesset A. (2008). General purpose molecular dynamics simulations fully implemented on graphics processing units. J. Comp. Phys..

[16] Glaser J., Nguyen T.D., Anderson J.A., Liu P., Spiga F., Millan J.A., Morse D.C., Glotzer S.C. (2015). Strong scaling of general-purpose molecular dynamics on GPUs. Comput. Phys. Commun..

[17] HOOMD—Blue Web Page. http://codeblue.umich.edu/hoomd-blue.

[18] Frenkel D., Smit B. (2001). Understanding Molecular Simulation.

[19] Tuckerman M.E. (2010). Statistical Mechanics: Theory and Molecular Simulation.

[20] Weeks J.D., Chandler D., Andersen H.C. (1971). Role of repulsive forces in determining he equilibrium structure of simple liquids. J. Chem. Phys..

[21] Engelhardt L., Schröder C., Winpenny R.E.P. (2011). Simulating Computationally Complex Magnetic Molecules. Molecular Cluster Magnets.

[22] Milstein G.N., Tretyakov M.V. (2004). Stochastic Numerics for Mathematical Physics.

[23] Dünweg B., Ladd A.J.C. (2009). Lattice Boltzmann simulations of soft matter systems. Adv. Polym. Sci..

[24] Batchelor G.K. (1976). Brownian diffusion of particles with hydrodynamic interaction. J. Fluid. Mech..

[25] Rosensweig R.E. (2014). Ferrohydrodynamics.

[26] Thomas S., Kalarikkal N., Stephan A.M., Raneesh B. (2014). Advanced Nanomaterials: Synthesis, Properties and Application.

[27] Teich L., Schröder C., Müller C., Patel A., Meyer J., Hütten A. (2015). Efficient calculation of low energy configurations of nanoparticle ensembles for magnetoresistive sensor devices by means of stochastic spin dynamics and Monte Carlo methods. Acta. Phys. Pol. A.

[28] Lingenheil M., Denschlag R., Reichold R., Tavan P. (2008). The “hot-solvent/cold-solute” problem revisited. J. Chem. Theory Comput..

[29] Car R., Parrinello M. (1985). Unified approach for molecular dynamics and density-functional theory. Phys. Rev. Lett..

[30] Marx D., Hutter J. (2009). Ab initio Molecular Dynamics: Basic Theory and Advanced Methods.

[31] Wiser N. (1996). Phenomenological theory of the giant magnetoresistance of superparamagnetic particles. J. Magn. Magn. Mater..

